# Structure-Based Mutational Analysis of eIF4E in Relation to *sbm1* Resistance to Pea Seed-Borne Mosaic Virus in Pea

**DOI:** 10.1371/journal.pone.0015873

**Published:** 2011-01-24

**Authors:** Jamie A. Ashby, Clare E. M. Stevenson, Gavin E. Jarvis, David M. Lawson, Andrew J. Maule

**Affiliations:** 1 John Innes Centre, Norwich Research Park, Norwich, United Kingdom; 2 Department of Biochemistry, University of Cambridge, Cambridge, United Kingdom; 3 School of Pharmacy, Queen's University Belfast, Belfast, United Kingdom; Ecole Normale Superieure, France

## Abstract

**Background:**

Pea encodes eukaryotic translation initiation factor eIF4E (eIF4E^S^), which supports the multiplication of *Pea seed-borne mosaic virus* (PSbMV). In common with hosts for other potyviruses, some pea lines contain a recessive allele (*sbm1*) encoding a mutant eIF4E (eIF4E^R^) that fails to interact functionally with the PSbMV avirulence protein, VPg, giving genetic resistance to infection.

**Methodology/Principal Findings:**

To study structure-function relationships between pea eIF4E and PSbMV VPg, we obtained an X-ray structure for eIF4E^S^ bound to m^7^GTP. The crystallographic asymmetric unit contained eight independent copies of the protein, providing insights into the structurally conserved and flexible regions of eIF4E. To assess indirectly the importance of key residues in binding to VPg and/or m^7^GTP, an extensive range of point mutants in eIF4E was tested for their ability to complement PSbMV multiplication in resistant pea tissues and for complementation of protein translation, and hence growth, in an eIF4E-defective yeast strain conditionally dependent upon ectopic expression of eIF4E. The mutants also dissected individual contributions from polymorphisms present in eIF4E^R^ and compared the impact of individual residues altered in orthologous resistance alleles from other crop species. The data showed that essential resistance determinants in eIF4E differed for different viruses although the critical region involved (possibly in VPg-binding) was conserved and partially overlapped with the m^7^GTP-binding region. This overlap resulted in coupled inhibition of virus multiplication and translation in the majority of cases, although the existence of a few mutants that uncoupled the two processes supported the view that the specific role of eIF4E in potyvirus infection may not be restricted to translation.

**Conclusions/Significance:**

The work describes the most extensive structural analysis of eIF4E in relation to potyvirus resistance. In addition to defining functional domains within the eIF4E structure, we identified eIF4E alleles with the potential to convey novel virus resistance phenotypes.

## Introduction

Plant recessive resistance to virus infection is relatively common. For members of the *Potyviridae* a number of these resistances have been defined molecularly and identified as individual or multiple members of the families of proteins involved in the eukaryotic translation machinery. Hence, allelic variation in genes for the translation factor eIF4E has accounted for differential susceptibility of their hosts to *Pepper mottle virus* (PepMoV), *Pepper vein mottle virus* (PMMV), *Potato virus Y* (PVY), *Tobacco etch virus* (TEV), *Lettuce mosaic virus* (LMV), *Barley mild mosaic virus* (BaMMV), *Barley yellow mosaic virus* (BaYMV), *Bean yellow mosaic virus* (BYMV), *Zucchini yellow mosaic virus* (ZYMV; [1]) and *Pea seed-borne mosaic virus* (PSbMV). The paralogous gene *eIF(iso)4E* has also been implicated in recessive resistance to *Turnip mosaic virus* (TuMV), TEV, and LMV in *Arabidopsis* through the analysis of single and combined null mutants (see other references in the recent review by [2] and, unusually, through resistance to *Chilli veinal mottle virus* (ChiVMV) in pepper being conferred by simultaneous point mutations in both genes [3].

**Figure 1:**
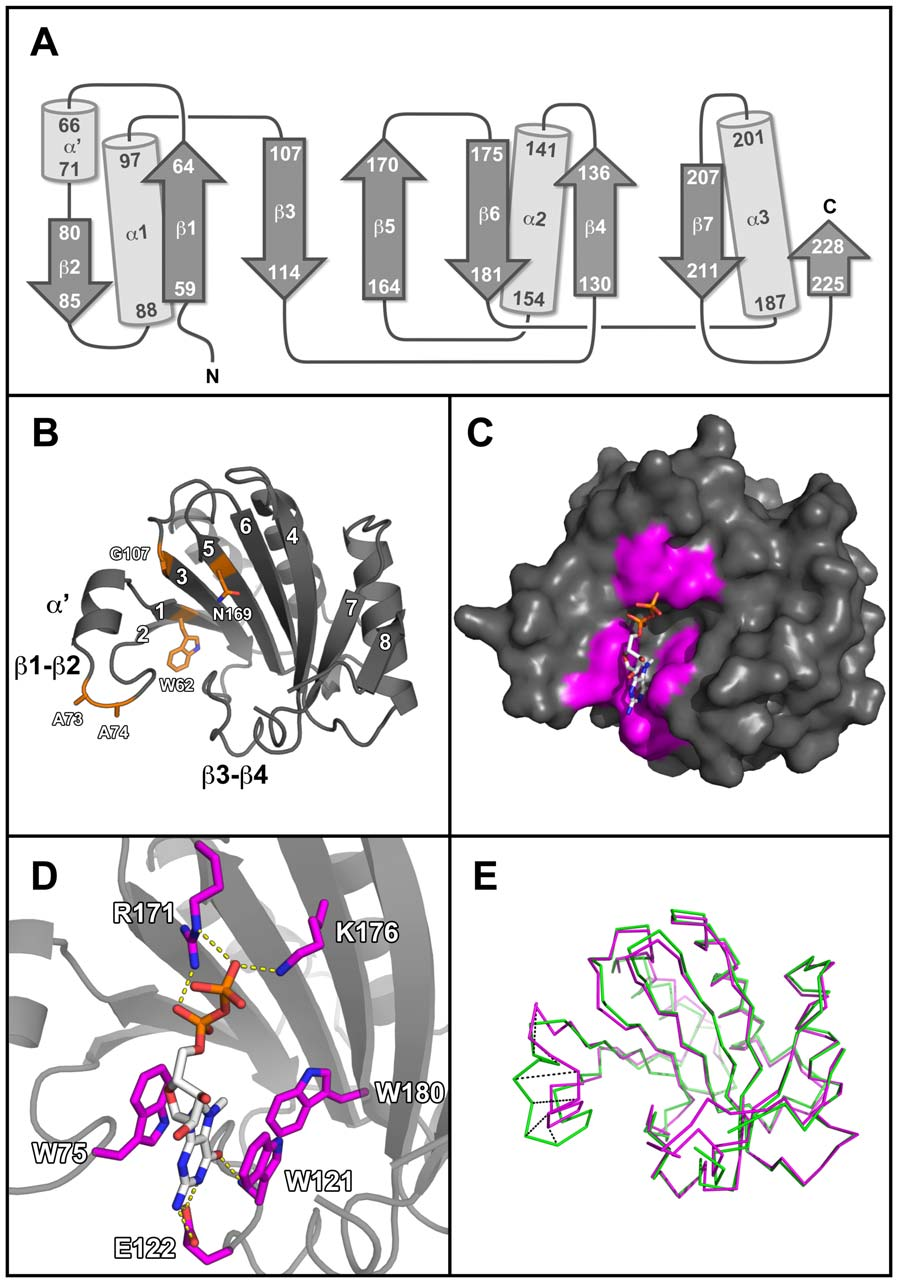
The structural organisation of pea eIF4E^ΔN51^. (A) Topology diagram depicting the secondary structure organisation of pea eIF4E^ΔN51^ derived from the PSbMV susceptible pea line JI2009. In contrast to previously reported eIF4E crystal structures from mammals, *Schistosoma* and wheat, pea eIF4E^ΔN51^ contains a short helical segment (α′) within the β1–β2 loop. (B) Cartoon representation of pea eIF4E^ΔN51^ chain H. The strands comprising the core β-sheet of the cap-binding site are labelled from one to eight and the position of the polymorphisms conferring *sbm1* resistance are coloured orange. (C) Surface representation of pea eIF4E^ΔN51^ chain H in complex with m^7^GTP. Residues interacting with the m^7^GTP cap analogue are coloured magenta. (D) Residues in pea eIF4E^ΔN51^ chain H making direct polar interactions with m^7^GTP. An additional van der Waals contact is made by the conserved W180 residue with the methyl group of m^7^GTP. The γ-phosphate of m^7^GTP was not visible in the electron density of any of the eight eIF4E molecules within the crystallographic asymmetric unit. (E) Superposition of the C_α_ backbones of pea eIF4E^ΔN51^ structure (chain H; green) and the wheat eIF4E^C113S^ mutant structure (PDB accession code 2IDV; magenta) giving a root mean square deviation of 0.708 Å over 171 structurally equivalent residues. Significant conformational differences between these orthologues can be observed in the β1–β2 loop and equivalent C_α_ atoms deviating by at least 5 Å between each structure are represented by dashed lines.

In pea, the *sbm1* resistance gene is effective against both BYMV [4] and a range of isolates of PSbMV [5] with lines carrying the dominant *SBM1* allele being universally susceptible to PSbMV, unless a second unlinked recessive resistance (*sbm2*) was present [6]. The *sbm1* gene was characterised as a mutant allele of pea eIF4E which differed from its wild type counterpart in five non-conservative amino acid substitutions [7] in the β1, β1–β2 loop, β3, and β5 regions, as defined for the crystal structure of pea eIF4E (Figure 1 A and B). Potyvirus resistance specificities from other plant species are similarly located proximal to these regions (reviewed in [2]). The extent to which these polymorphisms can confer resistance independently and the extent to which knowledge of individual mutations can be useful in selecting novel resistances across different plant species has not been comprehensively investigated. However, naturally occurring single polymorphisms in the pepper *pvr2^4^* (V67E; [8]), pepper *pvr1* (G107R; [9]), and lettuce *mo1^2^* (A70P; [10]) genes, and engineered single amino acid substitutions in the lettuce *mo1^0^* gene (W64A, F65A, W77L, R173A, W182A; [11]) do confer resistance to PVY in pepper, TEV in pepper and LMV in lettuce, respectively. In total, previously identified mutations associated with potyvirus resistance, found either alone or in combination [7], [8], [10], [12], [13], [14], [15], [16], are located on the β1, β1–β2 loop, α′, β3, β3–β4 loop, β4, β5, β5–β6 loop and α3-β7 loop secondary structures.

Resistance-breaking isolates of these viruses are not uncommon and comparative genetic and molecular analyses have identified the virus-genome linked protein (VPg) as the predominant avirulence factor [2]. This is supported by *in vitro* biochemical [12], [17], [18], [19] and yeast two-hybrid [8], [9], [12] assays that point to a direct interaction between VPg and the product of the dominant *eIF4E* susceptibility allele (eIF4E^S^), which does not occur with the resistance variant, eIF4E^R^. That other factors may be involved in the physical interaction, or its functional consequences, *in vivo* has been suggested from evidence for additional interactions with eIF4G [20] and for the involvement of further viral proteins: cylindrical inclusion protein (CI) and P1 protease have been implicated in overcoming eIF4E-based resistance in lettuce [21] and pea [22], respectively. Additional novel interactions between potyvirus helper-component proteinase (HC-Pro) and eIF4E or eIF(iso)4E from potato or tobacco have also been identified (J. Valkonen, personal communication). So far we have not been successful in demonstrating specific pea eIF4E-PSbMV VPg interactions *in vitro* (unpublished data) and have concluded that additional plant or viral factors may be required.

Crystal structures for mammalian [23], [24], [25], [26], [27], [28], *Schistosoma mansoni* [29] and wheat [30] eIF4E proteins have been published. They show a high degree of sequence and structural conservation. Moreover, these structures have been determined in complex with m^7^G cap analogues and thus revealed the molecular details of the cap-binding site. Based upon homology modelling [7], [9], [11], [12], [14], [31], the analysis of natural and engineered eIF4E variants [7], [8], [9], [10], [11], [12], [13], [14], [16], the interaction of eIF4E with potyvirus VPg in yeast [8], [9], and competition assays for binding of VPg and m^7^G analogues to eIF4E *in vitro* [17], [18], [19], [32], [33], [34], the location of the presumptive VPg binding site has been proposed to lie in two different regions of eIF4E; one is proximal to, and partially overlapping the interior of the cap binding pocket (region I), and another is on the surface of eIF4E facing 90° from the cap binding site (region II; [8], [30], [31]).

The potential for VPg interference in m^7^G cap analogue binding has focussed attention on viral RNA translation as the point in potyvirus replication supported by eIF4E^S^, and therefore, as a target for resistance in the presence of eIF4E^R^ (discussed in [31]). While this is consistent with the impact of VPg on translation *in vitro* [35] and with the correlation between eIF4E-VPg complex formation and virus infectivity [8], [18], it is not universally supported. Hence, internal translation initiation in the absence of eIF4E through direct binding of eIF4G or eIF4F at a putative internal ribosome entry site (IRES) on the 5′ untranslated region of the potyviral genome has been shown [32], [36], [37], [38], [39].

Recently, we have determined the crystal structure of eIF4E from pea [40] to provide a platform from which to test the structure-function relationships of the protein. In this paper we describe this structure and report the impact of a series of point mutations in and around the cap-binding pocket of pea eIF4E on infection with PSbMV, and use the protein crystal structure to provide a three-dimensional framework for interpreting these data. The conclusions support the view that the precise details of the interactions between particular eIF4Es and particular viral VPgs are highly specific and may not translate between different host-virus interactions. In addition, the data point to an incomplete overlap between eIF4E domains involved in translation and proposed VPg-binding, which suggests that the purpose of any protein-protein interaction may not be exclusively to support PSbMV RNA translation.

## Results and Discussion

### Crystal structure of pea eIF4E

In preparation for a structure-function analysis of pea eIF4E in relation to PSbMV susceptibility we determined the crystal structure of pea eIF4E^ΔN51^ by molecular replacement, using the wheat orthologue as a template [30]. Previous structural studies with other eIF4Es from mouse [41] and wheat [30] have shown that full-length protein can be recalcitrant to crystallization, whilst N-terminally truncated versions have been successfully crystallized. We therefore designed a version of the pea protein truncated at the position equivalent to that used for the crystallization of the wheat protein [30]. In support of this strategy, disorder prediction with the *FoldIndex* server (http:// bip.weizmann.ac.il/fldbin/findex; [42]) suggested that the first 51 residues of pea eIF4E are likely to be disordered. Furthermore, in tryptophan quenching assays (not shown), purified pea eIF4E^ΔN51^ retained m^7^GTP binding activity *in vitro*, indicating that the structural integrity of the truncated protein was not significantly compromised.

The X-ray data revealed eight independent copies of eIF4E in the crystallographic asymmetric unit, which assemble as two distorted tetramers, and within each tetramer a maximum of approximately 600 Å^2^ of protein surface is buried between pairs of subunits. Since only monomers were detected in solution [40], we conclude that these assemblies are simply crystal packing artefacts. More extensive dimer interfaces are present in both the mouse [25] and wheat [30] crystal structures, although both of these proteins were also monomeric in solution. Thus, the biologically relevant unit is most likely a monomer.

The monomer is comprised of a central 8-stranded anti-parallel β-sheet flanked by α-helices, although one face of the β-sheet is largely undecorated by α-helices and this is where the cap-binding site resides (Figure 1 C and D). The cap-binding pocket of eIF4E structures are characterised by a pair of tryptophan residues (W75 and W121 in the pea protein), which form π-stacking arrangements with the 7-methyl-guanine moiety (m^7^G) of the cap. These residues are located on the β1–β2 and β3–β4 loops, respectively, defining, in our views, the left and right hand sides of the pocket. The pea protein was co-crystallised with the cap analogue m^7^GTP. In all eight independent copies of eIF4E in the asymmetric unit (PDB accession code 2WMC; chains A–H) the cap-binding pocket is occupied with the ligand, although in none of these is the γ-phosphate visible in the electron density. The m^7^GTP ligand is further stabilised in the cap-binding site by a van der Waals contact with W180 on β5, and additional polar interactions with E122 on the β3–β4 loop, R171 on β5, and K176 on β6 (Figure 1 D).

In general, crystal structures provide a static image of a protein and much of the dynamic information is lost. However, the presence of eight independent copies of the molecule provides us with eight different snapshots of the pea eIF4E structure. By comparing these, we can begin to appreciate the dynamic behaviour of the molecule. In pairwise comparisons, the root mean square deviations vary between 0.415 and 1.414 Å for corresponding C_α_ positions. While the core β-sheet structure is essentially invariant, many of the surface loops and side-chains display multiple conformations. The β1–β2 loop displays significantly different conformations between the ligand-bound and ligand-free structures of the wheat orthologue [30]. In the pea structure, this is the most variable region of the whole structure and, in six out of the eight molecules, sections of it are not resolved at all due to weak electron density. Although in each case W75 is visible and maintains the π-stacking with m^7^GTP; the indole moiety adopts one of two different conformations that are related by a 180° flip about the C_β_-C_γ_ bond.

The pea structure most closely resembles that of the C113S mutant of the wheat protein with bound m^7^GDP (PDB accession code 2IDV), this being the top hit found by the Dali server ([43]; Z score 30.5). A superposition of the wheat structure with chain H of the pea structure gives a root mean square deviation of 0.708 Å over 171 structurally equivalent residues (Figure 1 E). The secondary structure of the two proteins is essentially the same although the pea protein has an additional short helical segment in the β1–β2 loop (termed α′, Figure 1 A and B). The two Cys residues that form a disulphide bridge in the wild-type wheat structure (PDB accession code 2IDR) are strictly conserved in plant orthologues. In the pea structure the two Sγ atoms are in close proximity (e.g. 4.4 Å apart in chain H), but are clearly not bridged. The biological significance of the disulphide bridge seen in the wheat structure remains uncertain.

### eIF4E-VPg interaction *in vivo*

**Figure 2:**
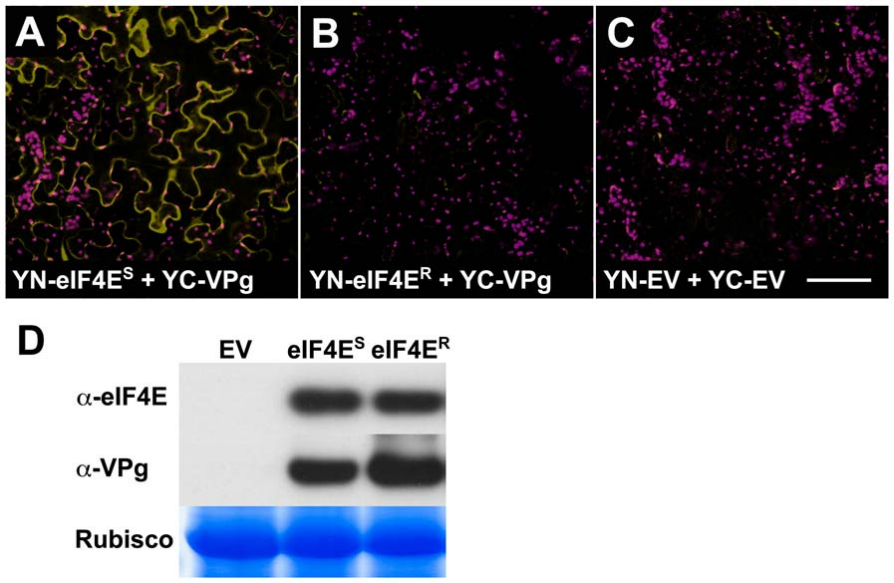
Pea eIF4E^S^ interacts with PSbMV VPg *in planta*. *N. benthamiana* leaves were co-infiltrated with constructs encoding TBSV P19 silencing suppressor [65] , PSbMV-P1 VPg fused to the C-terminal portion of YFP (YC-VPg) and either pea eIF4E^S^ or eIF4E^R^ fused to the N-terminal portion of YFP (YN-eIF4E). In control experiments, the equivalent empty expression vectors (YN-EV and YC-EV) were infiltrated in the presence of P19. YFP-specific fluorescence (yellow) and chloroplast autofluorescence (magenta) was recorded at 72 h post-infiltration by confocal microscopy. (A) A strong yellow fluorescence signal was detected in leaf epidermis following transient expression of YC-VPg and YN-eIF4E^S^ (B) Expression of the YC-VPg+YN-eIF4E^R^ combination resulted in a significantly lower yellow fluorescence signal. (C) Similar to that found for eIF4E^R^, yellow fluorescence was negligible following expression of the YN-EV+YC-EV vector controls. (D) Immunoblot analysis of total proteins confirmed that equivalent levels of eIF4E^S^, eIF4E^R^ and VPg were present in infiltrated tissue. Size bar = 100 µm. In A–C data are representative of three independent experiments.

In this work , we aimed to use mutagenesis to uncover features of the eIF4E-VPg interaction. Since, despite exhaustive experimentation, we had been unsuccessful in demonstrating such a direct interaction *in vitro*, we tested the partners for their ability to interact *in vivo* using bimolecular fluorescence complementation (BiFC; [44], [45]). For this, N- and C-terminal portions of yellow fluorescent protein (YN- and YC-YFP) were fused to the amino-termini of eIF4E^S^ and VPg, respectively. The constructs were expressed transiently in *Nicotiana benthamiana* using *Agrobacterium* as a delivery vehicle; empty vector controls were run in parallel (Figure 2 A and C). Since our hypothesis was that a physical interaction was necessary to support virus multiplication, potential interaction between VPg and the eIF4E^R^ resistance protein was also tested (Figure 2 B). Of these protein combinations, only YN- eIF4E^S^ and YC-VPg produced fluorescence *in vivo* (Figure 2 A). Immunoblot analysis of the expressed proteins showed that the absence of fluorescence for YN-eIF4E^R^/YC-VPg could not be attributed to reduced protein accumulation (Figure 2 D).

*N. benthamiana* is the favoured experimental host for agrobacterium-mediated transient expression and is susceptible to PSbMV. The positive physical interaction, recorded as BiFC, supports the evidence for physical interaction obtained in different potyvirus systems. The absence of a PSbMV infection in our experiments indicates that other viral proteins are not required for the physical interaction to occur. However, it does not rule out the potential for host protein involvement that we could not supplant into our *in vitro* studies. These host proteins would most probably be functional orthologues of the proteins from the pea host. So far, only eIF4G [20] has been implicated in such a role.

### Mutational analysis of eIF4E

**Figure 3:**
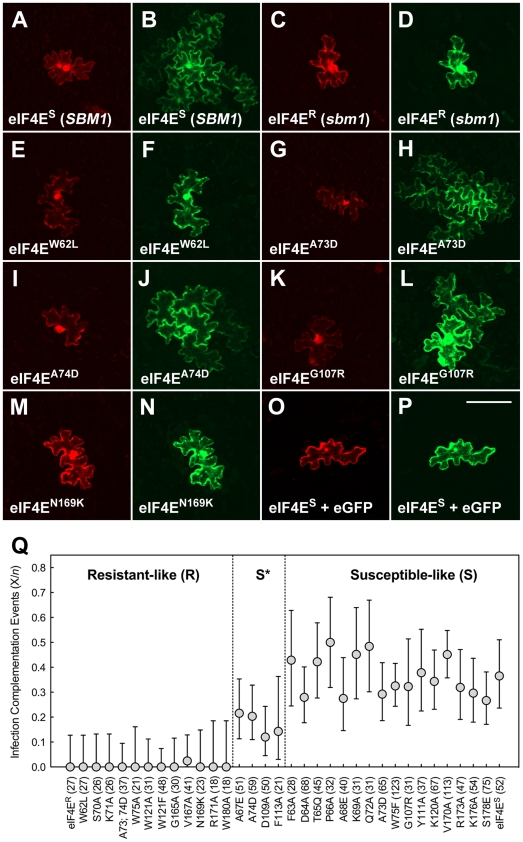
Complementation of PSbMV infection in resistant JI1405 pea. Leaf tissue of PSbMV resistant pea line JI1405 was biolistically co-bombarded with vectors encoding susceptible or mutant derivatives of mRFP-eIF4E together with PSbMV pathotype P1 expressing unfused GFP (PSbMV-P1.GFP). Primary foci (*n*) were recorded as single cells in which mRFP and GFP fluorescence could be observed and successful infection complementation events (X) were considered to be cases where GFP fluorescence was also detected in the neighbouring cells. (A–N) Representative images from the infection complementation assay corresponding to the polymorphisms collectively conferring *sbm1* resistance: (A and B) PSbMV-P1.GFP+mRFP-eIF4E^S^; (C and D) PSbMV-P1.GFP+mRFP-eIF4E^R^; (E and F) PSbMV-P1.GFP+mRFP-eIF4E^W62L^; (G and H) PSbMV-P1.GFP+mRFP-eIF4E^A73D^; (I and J) PSbMV-P1.GFP+mRFP-eIF4E^A74D^; (K and L) PSbMV-P1.GFP+mRFP-eIF4E^G107R^; (M and N) PSbMV-P1.GFP+mRFP-eIF4E^N169K^. (O and P) control assay in which JI1405 leaf was co-bombarded with susceptible mRFP-eIF4E^S^ (O) and a vector encoding eGFP (P) in the absence of PSbMV; eIF4E^S^ expression is insufficient to allow free GFP movement into neighbouring cells. Size bar = 100 µm. (Q) Graphical representation of the infection complementation data. eIF4E variants were plotted as the proportion of complementation events (X/*n*; *n* values are given in brackets). Statistical analysis (see Materials and Methods) classified the mutants as being ‘resistant-like’ (R), ‘partially-susceptible’ (S*) or ‘susceptible-like’ (S). Error bars represent the upper and lower 95% confidence limits.

In order to assess the behaviour of mutants in pea eIF4E with respect to PSbMV infection, we exploited a complementation assay used previously [7]. In this assay, co-bombardment of leaf tissue of *sbm1-*resistant pea (line JI1405) with cDNAs expressing eIF4E^S^ (from pea line JI2009), or its mutant derivatives, and PSbMV expressing unfused GFP (PSbMV-P1.GFP) resulted in complemented replication of the virus in the resistant cells that extended beyond the primary target into neighbouring cells. We concluded previously that this complementation in neighbouring cells reflected a non-cell-autonomous function for eIF4E in supporting PSbMV replication [7]. In the current mutagenesis experiments we aimed to use the large number of point mutants to separate genetically the complementing movement and replication functions. Unfortunately, ectopic expression of GFP from non-replicating PSbMV virus, mutated to encode both a Cys to Ala substitution within the catalytic domain of the NIa protease (equivalent to the TEV NIa^C151A^ mutant [46]) and a premature TAA termination sequence at the 3′-end of the VPg cistron, meant that we always observed fluorescence in primary target cells independent of the nature of the supporting eIF4E allele (data not shown). Nevertheless, virus replication in neighbouring cells was consistently observed at the majority of bombardment sites following complementation with eIF4E^S^ and was used as a measure of the effectiveness of mutant eIF4E derivatives to support virus multiplication (replication and movement). Data were recorded as the number of bombardment sites (visible as GFP fluorescence) for which virus replication (and therefore GFP fluorescence) in neighbouring cells was observed. All of the mutants were scored in at least three independent experiments. The data showed that the mutation of individual residues led mostly to quantitative rather than qualitative changes in the resistance response, ranging from those equivalent to the eIF4E^S^ and eIF4E^R^ controls to efficiencies higher than that observed for eIF4E^S^. Statistical analysis (see Materials and Methods) classified these activities as ‘susceptible-like’ (S), ‘resistant-like’ (R) or ‘partially susceptible’ (S*) in their ability to support PSbMV infection (Table 1; Figure 3).

**Table 1:** Summary of the biological properties of pea eIF4E mutants.

eIF4E variant	PSbMV infection	Yeast Translation	Conservation Score
eIF4E^S^	S	++	NA
eIF4E^R^	R	+	NA
W62L^a^	R	+	8
F63A	S	++	8
D64A	S	++	9
T65Q	S	++	7
P66A	S	++	5
A67E	S*	++	4
A68E	S	++	2
K69A	S	++	6
S70A	R	++	8
K71A	R	++	3
Q72A	S	++	8
A73D^a^	S	++	3
A73D;A74D	R	++	NA
A74D^a^	S*	++	4
W75A^b^	R	−	7
W75F^b^	S	+	7
G107R^a^	S	++	2
D109A	S*	++	7
Y111A	S	+	7
F113A	S*	−	9
K120A	S	++	8
W121A^b^	R	−	9
W121F^b^	R	−	9
E122A^b^	ND	−	9
G165A	R	−	9
V167A	R	++	9
N169K^a^	R	++	7
V170A	S	++	7
R171A^b^	R	−	9
R173A	S	++	5
K176A^b^	S	+	7
S178E	S	−	7
W180A^b^	R	−	9

a Mutation found in the *sbm1* resistance allele of pea eIF4E.

b Amino acid position involved in binding m^7^GTP in the pea eIF4E crystal structure.

ND = no data, NA = not applicable.

In the PSbMV infection complementation assay, eIF4E variants were classified as having a ‘susceptible-like’ (S), ‘partially-susceptible’ (S*) or ‘resistant-like’ (R) phenotype. The support of yeast translation was scored as full-growth (++), reduced growth (+) and abolished growth (−), relative to eIF4E^S^. Conservation scores represent the degree of amino acid conservation ranging from variable (1) to conserved (9) calculated for 68 non-redundant plant eIF4E sequences.

**Figure 4:**
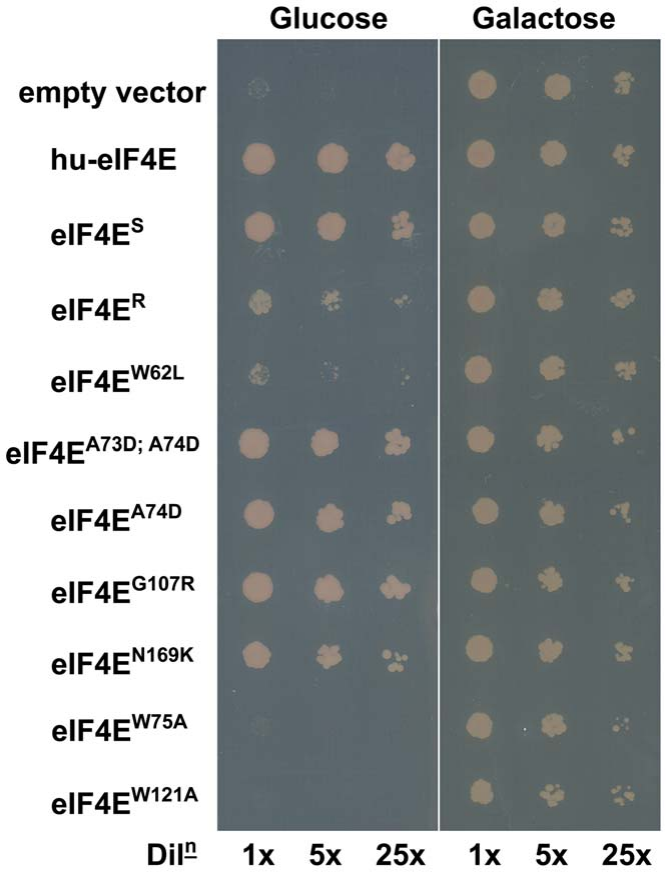
eIF4E-dependent rescue of yeast translation. Yeast strain Jo55 lacks a chromosomal eIF4E gene and only survives on galactose-containing media due to the presence of vector YCp33supex-h4E URA3, which encodes human eIF4E under the control of a glucose-repressible, galactose-dependent promoter. This strain was subsequently transformed with empty vector YCpTRP-GW (negative control), or the same vector containing the coding sequence of human eIF4E (positive control) and pea eIF4E derivatives under the control of the constitutive *TEF1* promoter. Transformed cells were grown in selective drop-out media (SD −Trp −Ura+galactose) to an optical density (OD_600_) of 1.0 before being serially diluted and assessed for growth on glucose- and galactose-containing media. Three independent experiments were performed for each eIF4E variant and translation complementation was scored as the relative level of cell growth. This ranged in amount from, for example, (++) for eIF4E^S^, (+) for eIF4E^R^ to no observable growth (**−**) for eIF4E^W75A^.

In parallel, expression of eIF4E^S^, and its mutant derivatives, was used to complement translation in an eIF4E-deficient yeast strain [47]. Although not a direct assessment of translational competence *in planta*, this assay provides a convenient measure of general competence for eukaryotic translation, including cap-binding, and is a good indicator of correct folding of the proteins under test. In accordance with previous work [11], translational efficiency was scored semi-quantitatively as full (++), reduced (+) or abolished (−) yeast colony growth on appropriate selective media following serial dilutions (Table 1; Figure 4). We acknowledge, however, that this yeast assay need not be fully representative of translational competence *in planta* and especially that the precise effects of individual mutations may differ.

Mutations to eIF4E^S^ were made as listed in Table 1 and included: (1) the individual polymorphisms present in eIF4E^R^ (from line JI1405; W62L, A73D, A74D, G107R and N169K); (2) mutations of the residues interacting with m^7^GTP in all eight molecules of the pea eIF4E asymmetric unit (W75, W121, E122, R171, K176, W180; Figure 1 C and D); and (3) a range of mutants proximal to the cap-binding site, a region in which single amino acid substitutions were previously demonstrated to result in potyvirus resistance [8], [9], [10], [11]. Collectively, these mutations were located on β1, β1–β2 loop (including α′), β3, β3–β4 loop, β5, β5–β6 loop and β6. In our crystal structure, there was no evidence for interactions between m^7^GTP and the α1, α2 or β4 structural features. Furthermore, limited mutational analysis of α1 and α2 [11] suggested that these structural elements are not major determinants for potyvirus infection. Therefore, the relative lack of exposure of these features to the cap-binding pocket gave them a lower priority in our analysis. These mutations in eIF4E comprise the most comprehensive set designed to test structure-function relationships in potyvirus resistance.

#### Mutations conferring *sbm1* resistance to PSbMV act combinatorially

As expected, the eIF4E^R^ protein was unable to complement PSbMV-P1.GFP infection in leaf tissue from resistant pea line JI1405 (Table 1). In yeast, expression of eIF4E^R^ resulted in only moderate cell growth, when compared to the action of eIF4E^S^, indicating that this protein was competent in supporting translation, albeit to a lower level. Of the individual polymorphisms conferring *sbm1* resistance, all except W62L fully supported yeast growth; W62L showed only weak growth. Whatever the impact of the W62L mutation in isolation, it seems possible that it could modulate translational efficiency to the lower level of activity seen in eIF4E^R^, when in combination with the other mutations. In the infection complementation assay, the A73D and A74D mutations were tested individually and in combination. Whereas A73D resulted in a susceptible-like phenotype (S), the A74D mutant displayed only partial activity (S*). Interestingly, the double mutation (A73D; A74D) showed no complementation activity. Of the other polymorphisms, only W62L and N169K displayed a full resistant-like (R) phenotype (Table 1). These data show that while single amino acid substitutions in pea eIF4E can significantly impact on PSbMV infection and yeast translation, the *sbm1* mutations collectively interact to produce a phenotype distinct from that conferred by each constituent polymorphism.

#### Mutations in residues involved in cap binding

The potential role of eIF4E cap-binding residues in potyvirus infection has been investigated [11]. This approach employed homology modelling to predict the position of such residues in eIF4E and prompted us to test the cap-binding residues we have identified in the pea eIF4E crystal structure for their ability to support translation in yeast and for their ability to support PSbMV replication/movement. With the exception of K176A, which displayed only weak translational competence (+), non-conservative substitutions to all other residues involved in cap-binding abolished translation in yeast (Table 1). The ability of this group of mutants to complement infection showed a similar pattern; with the exception of K176A, which successfully complemented infection, all of those tested were classified as being resistant-like (R). Both W75 and W121 form π-stacking arrangements with the m^7^G moiety of cap. Interestingly, restoration of an aromatic ring in the W75F mutant allowed limited translation (+) and resulted in a susceptible-like phenotype (S). This apparent gain of function did not extend to W121F, however, which was deficient in the complementation of both infection and yeast translation.

#### Mapping mutations affecting resistance/susceptibility

In addition to mutations related to the *sbm1* allele and the cap-binding residues, we targeted further sites based upon homology modelling of regions implicated elsewhere in potyvirus resistance and upon a more thorough analysis of residues in and around the cap-binding pocket. Subjecting this collective set of mutations to the complementation of virus multiplication assay *in planta* and translation assay in yeast gave rise to a number of phenotypic combinations (R/++, R/−, S/++, S/− etc). The largest proportion of mutants (12/33) was positive with respect to both virus multiplication and translation (S/++). Clearly, in the context of virus resistance the R/++ and R/+ classes are of interest, although the S*/+ and S*/− phenotypes also indicate less than wild type support for virus multiplication. Interpretation of the R/− class is difficult as these may represent defects in protein folding and stability. Mutants with S/− or S*/− phenotypes showed positive biological behaviour with respect to virus multiplication and therefore probably did not reflect major defects in protein folding.

**Figure 5:**
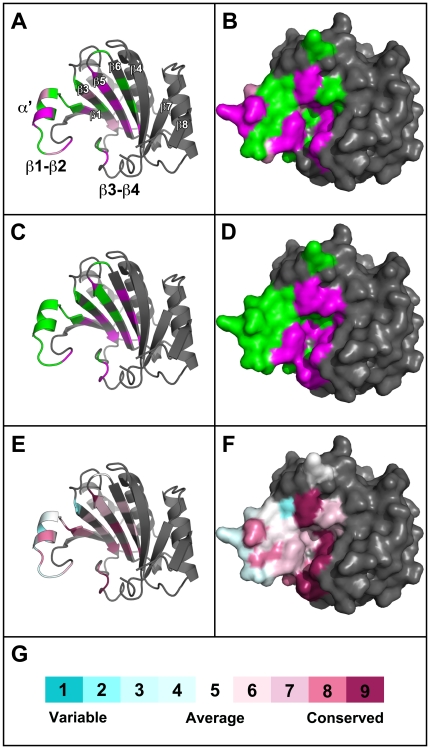
Mapping the biological properties of eIF4E mutants onto the pea crystal structure. (A and B) Results of the PSbMV infection complementation assay mapped onto chain H of the pea eIF4E^ΔN51^ crystal structure. (A) cartoon representation and (B) surface representation of eIF4E colour coded to depict the three classifications of infection complementation: susceptible-like (S; green), partially-susceptible (S*; pink) and resistant-like (R; magenta). (C and D) Results of the yeast translation complementation assay mapped onto chain H of the pea eIF4E^ΔN51^ crystal structure. (A) cartoon representation and (B) surface representation of eIF4E colour coded to depict the classifications of translation complementation: mutations resulting in full (++) and partial growth (+) are coloured green and those resulting in abolished growth (−) are coloured magenta. (E and F) Results of the evolutionary trace analysis mapped onto chain H of the pea eIF4E^ΔN51^ crystal structure. A sequence alignment was generated for the 68 non-redundant plant eIF4E sequences most closely related to pea eIF4E and was used to calculate the relative degree of evolutionary conservation at each amino acid position through an implementation of the Maximum Likelihood method (see Materials and Methods). A colour-coded scale (G) varying from 1 (highly variable) to 9 (fully conserved) was subsequently mapped onto the cartoon (E) and surface (F) representation of pea eIF4E in the amino acid positions included in the mutagenesis screen.

Five mutants (S70A, K71A, A73D;A74D, V167A, N169K) showed the R/++ phenotype, one mutant (W62L) the R/+ phenotype, and three mutants (A67E, A74D, D109A) the S*/++ phenotype. These mutations identify amino acid positions critical for PSbMV infection. The location of these residues are displayed on the eIF4E molecular model in Figure 5 (Panels A and B in magenta and pink, respectively). They are located in two general regions of eIF4E. In the first group, A67E, S70A and K71A lie on the α′ helix within the β1–β2 loop, and A74D is located proximal to the cap-binding residue W75 within the same loop. W62L lies at the end of β1 and is somewhat isolated from the other major resistance determinants; the closest being D109A (S*/++) whose C_α_ atom lies relatively distant to that of W62L at 8.3 Å, although both these residues have side chains facing into the cap-binding pocket. The last group of mutations are located close to the top (D109A, β3; N169K, β5) and central (V167A, β5) region of the cap-binding pocket (according to the orientation depicted in Figure 5). Broadly, these data confirm the distribution of determinants for natural and engineered eIF4E-based resistance to potyviruses and support the view that the physical location for binding of VPg overlaps with that for m^7^GTP. The β5 strand is, however, a novel location for determinants of any potyvirus resistance and may identify an important site for novel sources of resistance. Two alternative explanations are that it represents a host-specific adaptation not yet identified in pea germplasm or that its absence in the wider plant populations studied so far may also indicate that there are pleiotropic costs associated with such mutations.

The mutations on β5 may define the right-hand limit of VPg binding to the cap-binding pocket of pea eIF4E. K176 and S178 on β6 appear not to be involved in infection and no natural polymorphisms associated with potyvirus resistance map to this strand, or indeed on β4, further right of β6. The equivalent of W180A (bottom of β6) was engineered in lettuce eIF4E [11] and found to abolish LMV infection. However in our experiments, this mutation also abolished yeast translation, as did K176A, and S178E. We speculate that mutations on β6 may produce more general defects in eIF4E folding or structural integrity. The only resistance determinants known to map on β7 are for BaYMV (genus *Bymovirus*) infection in barley [15]. The impact of each constituent polymorphism in *rym5*-mediated resistance has not been determined, but this may indicate that, as with other plant viruses [48], more distantly related members of the *Potyviridae* have adapted to exploit eIF4E by alternative biochemical mechanisms.

We were unable to assay PSbMV VPg binding to pea eIF4E *in vitro*; extensive attempts by us to co-crystallise eIF4E in complex with VPg were also unsuccessful; VPg is known to be intrinsically disordered and is highly unstructured in solution [17], [49], [50]. Nevertheless, from BiFC following transient expression in *N. benthamiana*, we observed an eIF4E^S^-VPg interaction *in vivo*. Hence we propose that eIF4E mutants displaying the R/++, R/+ or S*/++ phenotypes are likely to represent amino acid positions important for VPg-binding.

With the exception of the mutants leading to inhibition of translation (R/−), for which a role in infection remains unclear because their inactivity in our assays might relate to problems with protein folding, other non-conservative substitutions in positions proximal to and directly neighbouring the R/++, R/+ and S*/++ mutants had little effect on the ability of eIF4E to support infection (Figure 5 A and B, coloured green). Considering the large number of mutants we have analysed, this would suggest that PSbMV infection is dependent on a rather limited number of eIF4E residues in defined positions, although we acknowledge that members of the R/− group may also be involved. In tomato, a single G107R substitution was sufficient to confer resistance to a range of TEV isolates [9]. Similarly, the pepper *pvr2^4^* allele, which differs from the susceptible *pvr2* ^+^ allele by a single polymorphism (A67E), led to PVY-LYE84 resistance in *Capsicum* [8]. In apparent contradiction to these findings, the equivalent G107R substitution in pea eIF4E resulted in full infection complementation, and the A67E mutation allowed a measurable, albeit reduced infection complementation activity.

Analysis of natural polymorphisms associated with eIF4E resistance in pepper identified changes associated with a number of relatively non-conserved residues [8], [12], [13]. These positions presumably allowed the evolution of a resistant genotype without incurring penalties associated with ancillary functions of eIF4E. Indeed, a range of resistance alleles from lettuce [11] and pepper [8] were shown to fully support eukaryotic translation in yeast. From our analysis, mutations important for virus multiplication were not restricted to non-conserved residues (e.g. W75; Table 1; Figure 5 E and F). However, six of the ten mutations leading to abolished infection also abolished translation in yeast. Although it remains plausible that paralogous activities of eIF(iso)4E may compensate for these dysfunctions *in planta*, growth defects have been described for an *Arabidopsis* mutant line lacking eIF4E [51] suggesting that mutations leading to the R/− phenotype may similarly affect pea development. Nevertheless it is conceivable that our R/++ or R/+ mutations would support translation in pea, and thus, represent good potential candidates for developing novel PSbMV resistances.

Overall, our data support the notion that although the residues on eIF4E required for infection physically overlap with the cap-binding site to some extent, PSbMV has adapted to utilise a defined set of eIF4E residues which are not necessarily involved in other eIF4E-potyvirus interactions, and that these residues provide candidates not currently identified within the limited survey of pea germplasm.

#### Analysing the relationship between translation and infection

The analogous properties of cap and VPg in binding to eIF4E have suggested a role for eIF4E in viral RNA translation, although this has been questioned (discussed in [31]). Notwithstanding possible differences in translation between yeast and host plants, the results from our mutagenesis of the cap-binding residues also suggested a possible link between the residues involved in translation and those involved in supporting PSbMV infection in pea. The analysis of additional point mutants located in and around the cap-binding pocket has allowed us to address this question in more depth. The mutations resided throughout the cap-binding pocket, including residues on the β1–β2 loop, and on and between the β1, β3, β5 and β6 strands with side-chains shown in the crystal structure to extend into the cap-binding pocket (Figure 1 A and B; 5 A–D). The results of our analysis were placed into two groups, broadly termed ‘coupled’ and ‘uncoupled’. The first group contained mutants that displayed any activity in the infection complementation assay (S or S* phenotypes) concomitant with any activity in the yeast rescue assay (++ or +), and also contained those mutants that displayed no activity in either assay (i.e. R/−). Conversely, the second group contained members that displayed any activity in one of the assays, but no activity in the other (R/++, S/− or S*/−). The data show that of the 31 single amino substitutions tested in both functional assays, 24 mutants belong to the ‘coupled’ group (77.4%) and seven mutants to the ‘uncoupled’ group (22.6%). Therefore, in agreement with the finding for LMV infection in lettuce [11], our analysis indicates that although there is a strong correlation between the two processes, the known roles of eIF4E in infection and translation can be functionally uncoupled. Most notable were two mutants, F113A (S*/−) and S178E (S/−) that showed no translation activity but some support for virus multiplication, albeit at a low level (Figure 3 Q). Thus, it would appear that although cap-dependent translation, as judged from the heterologous yeast assay,is not necessarily required for potentiating viral multiplication, the mechanisms by which these two processes operate are likely to be related structurally.

## Materials and Methods

### Plant and virus material

*Pisum sativum* L. (pea) line JI1405 (PSbMV-resistant) and *Nicotiana benthamiana* were grown in glasshouses with conditions of 14-h photoperiod/temperature of 18–22°C or 16h photoperiod/temperature 18–25°C, respectively. For virus infections and as a source of cDNA clones, PSbMV-P1.GFP was used [7].

### Plasmid construction

For the construction of *E. coli* expression vector pET-eIF4E^ΔN51^, the truncated eIF4E ORF was amplified by PCR from the eIF4E cDNA of *Pisum sativum* cultivar JI2009 (Genbank accession AY423375.2) and blunt-end cloned into pET-24a(+) (Novagen) previously digested with *Nde*I and made blunt with Klenow fragment. To construct the eIF4E mutants, the full-length eIF4E coding sequence was cloned into entry vector pDONR207 by recombination reactions using BP clonase II (Invitrogen).

Amino acid substitutions were subsequently introduced using QuikChange® site-directed mutagenesis (Stratagene). Following automated sequencing, entry clones were recombined into the appropriate destination vector using LR clonase II (Invitrogen). For expression of eIF4E in *S. cerevisiae*, vector YCpTRP-h4E [47] was made Gateway compatible by replacing the *Xba*I-*Xho*I fragment with the Gateway cassette of pDEST17 (Invitrogen), resulting in destination vector YCpTRP-GW. For functional complementation of infection, pDONR constructs containing eIF4E sequences were recombined with pB7WGR2,0 resulting in the pB7-mRFP-eIF4E series which express mRFP fused to the N-terminus of eIF4E. For BiFC assays, a pDONR207 construct containing the full-length sequence of PSbMV-P1 VPg was recombined with pGPTVII.Hyg.YC-GW [45], [52] resulting in the C-terminal portion of YFP being fused to the N-terminus of VPg (YC-VPg). For eIF4E, pDONR constructs containing full-length eIF4E sequences were recombined with pGPTVII-Bar.YN-GW resulting in the N-terminal portion of YFP being fused to the N-terminus of eIF4E (YN-eIF4E). The integrity of all constructs was verified by diagnostic restriction digest and automated sequencing. All primer sequences are available on request.

### Protein expression and purification

*E. coli* strain Rosetta-2 (DE3) pLysS (Novagen) was transformed with *E. coli* expression vector pET-eIF4E^ΔN51^ and cells were cultured in 1 L of LB medium containing 50 µg mL^−1^ kanamycin and 34 µg mL^−1^ chloramphenicol at 37°C with shaking. When an optical density (OD_600_) of ∼0.8 was reached, protein expression was induced with 0.4 m*M* isopropylthio-*β*-galactoside (IPTG) for 3 h at 21°C. Cells were harvested by centrifugation, resuspended in 15 mL buffer A (20 m*M* Hepes pH 7.6, 150 m*M* NaCl, 2 m*M* EDTA and 4 m*M* DTT) containing complete EDTA-free protease inhibitors (Roche Diagnostics) and disrupted by two passages through a French press before insoluble material was sedimented at 46,000 ***g*** for 30 min. Soluble eIF4E^ΔN51^ proteins were loaded onto a 3 mL m^7^GTP Sepharose 4B column (GE Healthcare) equilibrated with buffer A, washed with 20 column volumes of the same buffer and eluted with 100 µM m^7^GTP (Sigma Aldrich). Fractions containing the highest amount of eIF4E^ΔN51^ were pooled and further purified by gel filtration on a HiLoad 16/60 Superdex 75 column (GE Healthcare) in buffer B (20 m*M* Tris-Cl pH 7.6, 300 m*M* NaCl and 5 m*M* DTT). For storage, a final concentration of 2 m*M* EDTA and 800 µ*M* m^7^GTP was added and protein aliquots were rapidly frozen in liquid nitrogen and stored at −70°C.

### Protein Crystallisation and molecular modelling

eIF4E^ΔN51^ was crystallised and native X-ray data were collected to a maximum resolution of 2.2 Å as described [40]; data collection statistics are summarised in Table 2. The space group was P2_1_ with cell parameters of *a* = 73.61, *b* = 136.32, *c* = 74.41 Å, β = 92.65°. The crystal structure of the equivalent fragment of the orthologue from wheat was used as a template for molecular replacement (PDB accession code 2IDV), with which the pea protein shares 71% amino acid sequence identity over this region (60% identity overall). A molecular replacement search model was subsequently created from the wheat structure using the program *CHAINSAW* [53], [54] with reference to an alignment of the wheat and pea sequences generated using the CLUSTALW server [55], [56]. Molecular replacement was performed using the program *AMoRe* [57]. This was initially successful in finding six independent molecules in the asymmetric unit. Inspection of the crystal packing using the molecular graphics program *COOT* [58] revealed that there was space for additional molecules. However, attempts to find these with *AMoRe* gave unacceptable clashes with the existing molecules. Nevertheless, through the application of crystallographic and translational symmetry it was possible to rearrange the molecules as two groups of three, with each group forming three-quarters of a distorted C_4_ tetramer. The missing monomer from each tetramer was then located by extrapolation using the program *SUPERPOSE* [59]. The resultant assembly of two tetramers gave sensible crystal packing with no interpenetration of neighbouring molecules. The solvent content based on eight molecules per asymmetric unit was estimated at 46.1%. This starting structure was then subjected to 10 cycles of rigid body refinement with the program *REFMAC5* [60] to give R_cryst_ and R_free_ values of 36.9 and 37.2%, respectively. Subsequently, manual rebuilding of this model was performed with *COOT*, with reference to *SIGMAA*-weighted [61] 2mF_obs_ – DF_calc_ and mF_obs_ – DF_calc_ Fourier electron density maps, and this was alternated with restrained refinement with *REFMAC5* using one TLS domain per protein chain. The protein was co-crystallised with m^7^GTP and one nucleotide was clearly bound to each molecule. However, the γ-phosphate was not visible in the electron density for any of these, either due to disorder of this moiety or hydrolysis to give m^7^GDP. The parameters of the final model are summarised in Table 2.

**Table 2:** Summary of X-ray data and model parameters.

**Data collection**	
Resolution range^a^ (Å)	21.93–2.20 (2.32–2.20)
Unique reflections	72233 (10355)
Completeness^a^ (%)	97.4 (95.4)
Redundancy	3.7 (3.5)
R_merge_ a *^,^* b	0.067 (0.241)
<I>/<σI>^a^	14.6 (5.8)
Wilson B value (Å^2^)	23.5
**Refinement**	
R_cryst_ c (based on 95% of data; %)	18.0
R_free_ c (based on 5% of data; %)	25.0
Coordinate error^d^ (Å)	0.302
Ramachandran most favoured^e^ (%)	97.4
Ramachandran outliers^e^	2
rmsd bond distances (Å)	0.011
rmsd bond angles (°)	1.481
**Contents of model (molecules/non-hydrogen atoms)**	
Protein (residues/atoms)	1289/10788
m^7^GTP (molecules/atoms)	8/232
Waters	790
**Average temperature factors** f ** (Å^2^)**	
Main-chain atoms	22.6
Side-chain atoms	22.7
m^7^GTP	32.8
Waters	26.0
Overall	23.1
**PDB accession code**	2WMC

a The figures in brackets indicate the values for outer resolution shell.

b R_merge_ = Σ_h_ Σ_l_ |I_hl_−<I_h_>|/Σ_h_ Σ_l_ <I_h_>, where I_l_ is the l^th^ observation of reflection h and <I_h_> is the weighted average intensity for all observations l of reflection h.

c The R-factors R_cryst_ and R_free_ are calculated as follows: R = Σ(| F_obs_−F_calc_ |)/Σ| F_obs_ |×100, where F_obs_ and F_calc_ are the observed and calculated structure factor amplitudes, respectively.

d Estimate of the overall coordinate errors calculated in *REFMAC5* [60].

e As calculated using *MOLPROBITY* [67].

f From *TLSANL* [68] output.

### Evolutionary trace analysis

Amino acid conservation scores were calculated using the ConSurf Server (http://consurf.tau.ac.il/index.html) [62], [63]. The UniProt database was queried with the pea eIF4E^S^ sequence (pea line JI2009; GenBank accession AY423375.2) using the *PSI-BLAST* program [64]. The top 68 non-redundant plant eIF4E sequences were aligned using *ClustalW* and conservation scores were calculated using the Maximum Likelihood method. ConSurf colour-coded conservation scores (Table 1, Figure 5 E–G) were subsequently mapped onto the pea eIF4E^ΔN51^ (chain H) crystal structure using *PyMOL* software (DeLano Scientific, CA, USA).

### Bimolecular Fluorescence Complementation (BiFC)

*Agrobacterium tumefaciens* strain GV3101 carrying the BiFC constructs of interest and one carrying a p19 silencing suppressor encoding plasmid [65] were brought to an OD_600_ of 0.5 with 10 mM MgCl_2_ and 150 mM acetosyringone (Sigma Aldrich). The strains were mixed and incubated at room temperature for 2 h and infiltrated into *N. benthamiana* leaves as previously described [65], [66]. Reconstituted YFP fluorescence was visualised 72 h after infiltration using a Zeiss 510 Meta confocal microscope. An argon laser at 488 nm was used for excitation with a 515 nm beam splitter and the spectral detector was set between 510 and 550 nm. Images were processed with Adobe Photoshop CS software (Adobe Systems, Inc.). Western blot analysis was performed to validate the stability of the protein fusions. Total proteins were extracted from infiltrated *N. benthamiana* leaf tissue in 50 m*M* Tris-Cl pH 6.8 containing 1% (w/v) SDS and 1 m*M* EDTA. Samples were precipitated with 70% (v/v) cold acetone and pelleted proteins were resuspended in the above buffer and quantified against known amounts of BSA using a BCA protein assay (Thermo Scientific). Equal amounts of total protein were separated by 12% SDS-PAGE before replicate gels were either stained with Instant Blue protein stain (Expedeon) or blotted onto PVDF membrane. Membranes were probed with anti-eIF4E antiserum (raised in rabbits against purified eIF4E^ΔN51^ protein) for the detection of YN-eIF4E fusions and anti-VPg-Pro (raised to *Potato virus A* VPg; gift from Prof J. Valkonen) to detect the YC-VPg fusion, respectively.

### Yeast translation complementation assays

*Saccharomyces cerevisiae* strain Jo55 (*cdc33*-Δ::*LEU2 leu2 ura3 his3 trp1 ade2* [YCp33 Supex2-hu4E:*URA3*] Gal^d^) lacks an endogenous chromosomal copy of the eIF4E gene (*cdc33*-Δ) and survives due to the presence of plasmid YCp33 Supex2-hu 4E:*URA3* which expresses a human eIF4E cDNA under the control of a glucose-repressible, galactose-dependent promoter [47]. The coding sequences of human eIF4E, pea eIF4E^S^ and pea eIF4E mutant variants were introduced into the Trp-selectable *E. coli*-yeast shuttle vector YCpTRP-GW for constitutive expression under the *TEF1* promoter. Following transformation of yeast strain Jo55, transformants were selected on galactose-containing media and grown to an optical density (OD_600_) of 1.0. Pelleted cells were resuspended in distilled water and serially diluted before eIF4E-dependent translation complementation was assessed on selective drop-out media containing glucose. Each experimental condition was performed in triplicate and the degree of yeast growth was assigned a score relative to the level of growth supported by eIF4E^S^.

### Functional complementation of PSbMV infection

PSbMV-P1.GFP [7] and pB7mRFP-eIF4E expression vectors were coated onto gold particles (1 µm diameter; BioRad) at a ratio of 1∶1 and used to inoculate the second and third detached leaves of pea seedlings in a Biolistic PDS-1000/He particle delivery system (BioRad). Following bombardment, leaves were maintained on MS agar (2% w/v) at 23°C with a photoperiod of 16 h. Inoculated cells were analysed at 72 h post bombardment for the presence of GFP and mRFP-eIF4E using a Zeiss 510 Meta confocal microscope.

For each mRFP-eIF4E mutant, a count (*n*) was made of the initially transfected cells in which both GFP and mRFP fluorescence was observable. Of these cells, the number of cases in which GFP fluorescence was also observed in cells adjacent to the primary foci (X) was used to calculate the proportion of successful infection complementation events (X/*n*). Due to variability in the efficiency of transfection, data were pooled from at least three independent replicate experiments. An initial analysis was performed using a 34×2 contingency table and the null hypothesis stating that the number of complementation events is the same for each eIF4E mutant was rejected (P = 6×10^−28^). Comparison of individual mutants required the comparison of multiple proportions. Since variables which are proportions are not normally distributed, the data were first transformed using a modified arcsine transformation after which a procedure analogous to Tukey's multiple comparisons was used. A *P* value less than 5% was considered significant.

The analysis distinguished three groups of mutants: (1) those mutants with X/*n* values significantly different from the susceptible *SBM-1* allele encoding eIF4E^S^ (pea line JI2009; X/*n* = 0.37). Mutants within this group were termed ‘resistant-like’ (R); (2) those mutants with X/*n* values significantly different from the *sbm-1* allele encoding eIF4E^R^ (pea line JI1405; X/*n* = 0.0). Mutants within this group were termed ‘susceptible-like’ (S); (3) those mutants with X/*n* values not significantly different from either JI2009 (susceptible) or JI1405 (resistant) pea lines. Mutants within this group were termed ‘partially susceptible’ (S*; Table 1, Figure 3 Q, 5 A and B ).
